# Specific centiloid thresholds to describe the continuum of amyloid and tau deposition

**DOI:** 10.1002/alz70856_102938

**Published:** 2025-12-24

**Authors:** Gregory Mathoux, Cecilia Boccalini, Débora E. Peretti, Giovanni B. Frisoni, Valentina Garibotto

**Affiliations:** ^1^ Geneva University Hospitals, Geneva, Switzerland; ^2^ University of Geneva, Geneva, Switzerland, Switzerland; ^3^ Laboratory of Neuroimaging and Innovative Molecular Tracers (NIMTlab), Geneva University Neurocenter and Faculty of Medicine, University of Geneva, Geneva, Switzerland; ^4^ Geneva Memory Center, Department of Rehabilitation and Geriatrics, Geneva University Hospitals, Geneva, Geneva, Switzerland; ^5^ Laboratory of Alzheimer's Neuroimaging and Epidemiology (LANE), Brescia, Italy; ^6^ Centre for Biomedical Imaging (CIBM), Geneva, Switzerland; ^7^ Faculty of Medicine, University of Geneva, Geneva, Geneva, Switzerland; ^8^ Division of Nuclear Medicine, Geneva University Hospitals, Geneva, Switzerland

## Abstract

**Background:**

The Centiloid (CL) scale standardizes amyloid PET imaging for consistent measurements across multicenter studies. However, varying thresholds for amyloid positivity create challenges in identifying individuals at risk for cognitive decline and specific pathological profiles. This study aims to define optimal CL thresholds using a three‐level classification system and validate their relevance in distinguishing tau pathology, cognitive status, and clinical outcomes in a memory clinic cohort.

**Method:**

A total of 580 participants, ranging from cognitively unimpaired to those with dementia, underwent amyloid‐PET scans ([18F]florbetapir or [18F]flutemetamol). Subsets underwent [18F]Flortaucipir‐PET (*n* = 185) and CSF biomarker analysis (*n* = 190). Participants were classified into three categories based on predefined thresholds: NEGATIVE (CL<12), GRAY‐ZONE (12≤CL≤37), and POSITIVE (CL>37). Associations between amyloid categories, tau status, CSF biomarkers, and cognitive performance were evaluated with Chi‐squared and Kruskal‐Wallis tests, while prognostic implications were assessed using linear mixed‐effects models. ROC analyses and Youden's Index were applied to determine additional CL thresholds for tau pathology and cognitive decline.

**Result:**

Predefined thresholds stratified participants effectively: 99% of POSITIVE individuals were visually amyloid‐positive, while 44% of GRAY‐ZONE individuals showed discordant visual classification. CSF levels decreased significantly across groups, with NEGATIVE individuals showing the highest and POSITIVE individuals the lowest levels (*p* <0.01). GRAY‐ZONE individuals exhibited intermediate characteristics, with higher tau burden and faster cognitive decline than NEGATIVE individuals (*p* <0.01), but less severe than POSITIVE individuals (*p* <0.01). ROC analyses identified optimal CL thresholds for specific outcomes. A threshold of 13 CL distinguished stable individuals from decliners (AUC=0.677), and thresholds of 14 and 51 CL accurately distinguished tau‐positive from tau‐negative cases (AUC>0.841). Lower threshold were associated with early mesial temporal tau positivity, while higher threshold indicated advanced tau pathology.

**Conclusion:**

Our findings demonstrate the importance of moving beyond binary amyloid classification and identify at least 3 meaningful levels. The GRAY‐ZONE group shows distinct clinical and biomarker profiles, with lower CL thresholds identifying individuals at higher risk for cognitive decline and early mesial temporal tau deposition, while higher CL values pinpoint individuals with amyloid and advanced tau pathology. Properly identifying these individuals is crucial for personalized treatment strategies, particularly as amyloid‐targeting therapies become more widely accessible.

